# The role of intraoperative radiation therapy in resectable pancreatic cancer: a systematic review and meta-analysis

**DOI:** 10.1186/s13014-020-01511-9

**Published:** 2020-04-09

**Authors:** Liang Jin, Ning Shi, Shiye Ruan, Baohua Hou, Yiping Zou, Xiongfeng Zou, Haosheng Jin, Zhixiang Jian

**Affiliations:** Department of General Surgery, Guangdong Provincial People’s Hospital, Guangdong Academy of Medical Sciences, Guangzhou, 510080 China

**Keywords:** Resectable pancreatic cancer, Intraoperative radiotherapy, Median survival time, Local recurrence, Postoperative complications and operation-related mortality

## Abstract

**Purpose:**

Several studies investigating the role of intraoperative radiotherapy (IORT) in the treatment of resectable pancreatic cancer (PC) have been published; however, their results remain inconsistent. By conducting a systematic review and meta-analysis, this study aimed to compare clinical outcomes in patients with resectable PC who underwent surgery with or without IORT.

**Methods and materials:**

The MEDLINE/PubMed, EMBASE, and Cochrane Library databases were searched to identify relevant studies published up to February 28, 2019. The main outcome measures included median survival time (MST), local recurrence (LR), postoperative complications, and operation-related mortality. Pooled effect estimates were obtained by performing a random-effects meta-analysis.

**Results:**

A total of 1095 studies were screened for inclusion, of which 15 studies with 834 patients were included in the meta-analysis. Overall, 401 patients underwent pancreatic resection with IORT and 433 underwent surgery without IORT. The pooled analysis revealed that IORT group experienced favorable overall survival (median survival rate [MSR], 1.20; 95% confidence interval [CI], 1.06–1.37, *P* = 0.005), compared with patients who did not receive IORT. Additionally, the pooled data showed a significantly reduced LR rate in the IORT group compared with that in the non-IORT group (relative risk [RR], 0.70; 95% CI, 0.51–0.97, *P* = 0.03). The incidences of postoperative complications (RR, 0.95; 95% CI, 0.73–1.23) and operation-related mortality (RR, 1.07; 95% CI, 0.44–2.63) were similar between the IORT and non-IORT groups.

**Conclusion:**

IORT significantly improved locoregional control and overall survival in patients with resectable PC, without increasing postoperative complications and operation-related mortality rates.

## Introduction

Pancreatic cancer (PC) is associated with poor clinical outcomes [1]. Surgical resection remains the mainstay therapy for PC; however, < 20% of patients are candidates for resection [2]. Moreover, even if a curative resection is performed, the 5-year survival remains relatively low [3–6]. Similarly, considering the high rate of postoperative local recurrence (LR) [7], most patients with PC will die because of local progression.

Recently, chemotherapy achieved some impressive advancements in treatment of PC [8, 9]. Due to the progression in multidisciplinary therapy, the ability of local control may play increasingly important role in improving survival for PC. Fortunately, with the electron beam technique that became available in the past few decades [10, 11], intraoperative radiotherapy (IORT) has provided an effective method as part of multidisciplinary therapy in resectable PC.

The feasibility of IORT in cancer treatment was reported as early as 1905 by Comas and Prio [12]. IORT is a safe and effective procedure that can be administered without the risk of additional morbidity or mortality, and it has led to considerable improvement of local control [13]. However, other reports suggested that IORT was associated with more serious postoperative complications and was not associated with increased overall survival [14, 15]. To date, the effect of IORT on long-term outcomes of resectable PC has not been completely determined. Thus, this meta-analysis aimed to compare the long-term outcomes of IORT versus non-IORT in patients with resectable PC.

## Methods and materials

This meta-analysis was conducted according to the Preferred Reporting Items for Systematic Reviews and Meta-Analyses guideline [16].

### Data sources and search strategy

We performed a computerized search of English-language publications listed in the electronic databases of MEDLINE/PubMed, EMBASE, and Cochrane Library to identify relevant studies published up to February 28, 2019. The following text and key words were used in combination to identify the studies: “pancreas cancer” and “intraoperative” and “radiotherapy.” The full search strategy is provided in the Supplementary Material (File 1).

### Inclusion and exclusion criteria

Studies included in our analysis had to (1) include resectable PC (without being metastatic or locally advanced); (2) include histologically proven PC; (3) contain two comparative groups (IORT vs. non-IORT); and (4) report the primary endpoints of the present meta-analysis (MST and LR). When several studies were reported by the same institution and/or authors, the publication with either the most complete data or that containing data with highest quality was selected for the meta-analysis. We excluded (1) studies wherein patients underwent palliative surgery; (2) studies reporting significant differences (*P <* 0.05) in tumor stages between the two comparative groups; (3) reviews without original data, duplicated publications, and animal studies; and (4) case reports, conference abstracts, review articles, and editorials.

### Study selection and outcome measures

Articles were reviewed and cross-checked independently by two authors (LJ, NS). The title or abstract of studies initially selected by a systematic search were preliminarily screened, and then, full texts of potentially suitable studies were reviewed according to the inclusion criteria. Data on the following characteristics were independently extracted: author identification, publication year, study country, study design, study period, number of patients, and characteristics of the study population. The corresponding authors were contacted to verify the extracted data and to request for additional data if the required information was unavailable from the published article. Any discrepancies were resolved by a third investigator (XZJ) and were confirmed by consensus.

Two reviewers (LJ, NS) independently evaluated the quality of the observational studies using the Newcastle-Ottawa Scale criteria [17]. We developed the evaluation criteria using score ranging from 0 to 9 points for cohort and case-control studies, with higher score corresponding to a higher study quality. The primary endpoints of the present study were MST and LR, and the secondary endpoints were postoperative complications and operation-related mortality.

### Statistical analysis

This meta-analysis was performed according to the recommendations of the Cochrane Collaboration and Meta-analysis of Observational Studies in Epidemiology guidelines [18]. Relative risks (RRs) were used to quantify the primary and secondary study observation endpoints. Summary RRs (95% confidence interval [CI]) were calculated by pooling study-specific estimates using a random-effects model that included between-study heterogeneity because significant heterogeneity was anticipated among studies. We calculated the *I*^*2*^ (95% CI) statistics to assess heterogeneity across studies, applying the following interpretation for *I*^*2*^: < 50% = low heterogeneity; 50–75% = moderate heterogeneity; and > 75% = high heterogeneity [19]. The primary endpoint (MST) was analyzed using median survival ratio (MSR), which was the ratio of the median survival time between the two treatment groups [20, 21].

Subgroup analyses were performed to investigate potential sources of between-study heterogeneity. Publication bias, which was indicated with *P* values < 0.10, was assessed using funnel plots, and the tests were developed by Egger and Begg. All statistical analyses were performed using the Stata software, version 12.0 (STATA Corporation, College Station, TX, USA). Statistical analyses were two-sided, and *P* values < 0.05 were considered statically significant.

## Results

### Characteristics of included studies

**Fig. 1:**
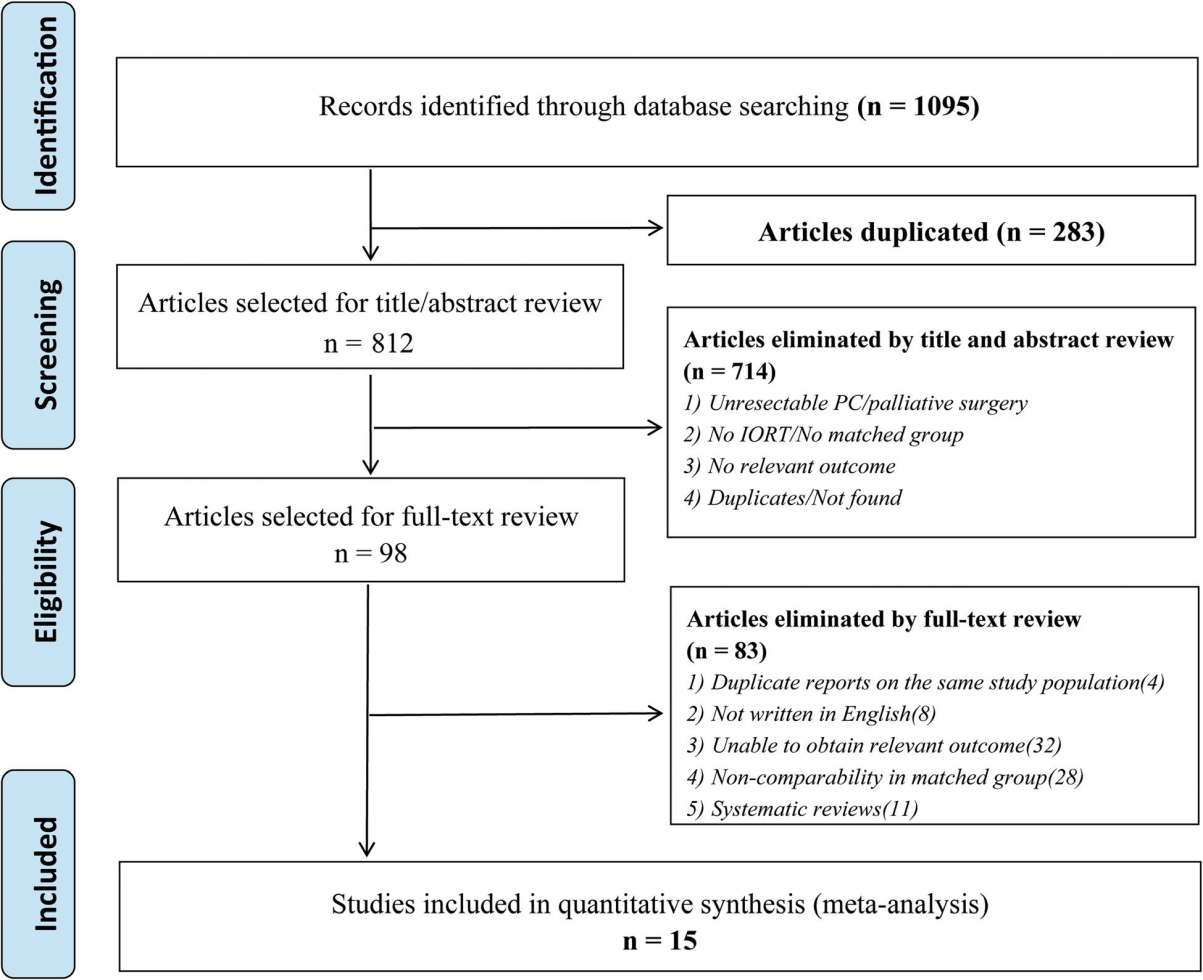
Flow diagram of the literature search according to PRISMA guidelines

Using the search strategy, 1095 unique articles were initially retrieved, of which 98 were considered of interest. The full texts of the 98 articles were retrieved for detailed evaluation, and finally, 15 studies complying with the inclusion criteria were assessed (Fig. 1).

Fifteen independent eligible studies [10, 14, 15, 22–33] were included in the analysis, these studies included 834 patients who underwent pancreatic resection with IORT (*n* = 401) or without IORT (*n* = 433). The studies were published between January 1971 and February 2019. There was one prospective study, 12 retrospective case-matched studies, and two studies that used a prospectively collected database. Based on whether patients underwent neoadjuvant or adjuvant treatment, CHT, or external beam radiotherapy (EBRT), these studies were divided into two subgroups: surgery (S) + IORT + EBRT + CHT versus S + EBRT + CHT (*n* = 6) and S + IORT versus S alone (*n* = 12). The Dobelbower et al. [14] study contained more than two subgroups (the Dobelbower-1 represented S + IORT + EBRT + CHT vs. S + EBRT + CHT subgroup, the Dobelbower-2 represented S + IORT vs. S alone subgroup). Koukubo and Shibamoto et al. [15, 26] reported their results according to R0 and non-R0 resection respectively (Koukubo-1 and Shibamoto-1 represented R0 resection, Koukubo-2 and Shibamoto-2 represented non-R0 resection).

The Newcastle-Ottawa Scale score involving 15 studies was ≥7, for the other four studies the score was equal to 6, indicating that all of the included articles had high quality [34]. All of the included studies described independent, consecutive sampling of their cohorts. Characteristics of the included studies are listed in Tables 1 and 2 (For additional details see Tables 4 and 5 in Electronic Supplementary Material).

**Table 1:** Characteristics of included studies comparing S + IORT+EBRT+CHT versus S + EBRT+CHT

Study	Nation	Study design	Study period	Sample size, n		Mean age (year)		Male, %		CHT, n		EBRT, n		Tumor Size (mean, cm)		Median Survival Time (month)		Tumor Staging, n (+) vs. (−)	Study quality
				(+)	(−)	(+)	(−)	(+)	(−)	(+)	(−)	(+)	(−)	(+)	(−)	(+)	(−)		
**Dobelbower-1 1997** [14]	USA	R	1980–1995	10	14	58.4	60.5	54.5%^a^		9	9	10	14	4.8	3.3	17.5	14.5	Stage I: 1 vs. 4 Stage II: 4 vs, 6 Stage III: 6 vs. 4	*******
**Nishimura 1997** [28]	Japan	R	1980–1995	32	24	62^a^		NA	NA	6	2	19	24	NA	NA	15.5	13.0	Stage I: 13% vs. 8% Stage II: 13% vs. 29% Stage III: 48% vs. 39% Stage IV: 28% vs. 24%	******
**Reni 2001** [10]	Italy	R	1985–1998	127	76	61.8	62.3	52	63.2	56	26	41	15	3.2	3.5	14.5	12.0	Stage I: 5 vs. 4 Stage II: 25 vs, 15 Stage III: 55 vs. 35 Stage IV: 42 vs. 22	*******
**Showalter 2009** [25]	USA	PCD	1995–2005	37	46	64	67	NA	NA	26	27	23	29	NA	NA	19.2	21.0	Stage I: 7 vs. 16 Stage IIA: 6 vs. 12 Stage ≥IIB: 24 vs. 18	******
**Calvo 2013** [22]	Spain	PCD	1995–2012	29	31	60	62	58.6	64.5	18	19	29	31	NA	NA	NA	NA	Stage IB-IIA: 13 vs. 16 Stage IIB-III: 16 vs. 15	*******
**Keane 2018** [29]	USA	R	2010- 2015	22	19	63^a^		37^a^		22	19	22	19	3.6^a^		35.1	24.5	41 underwent resection (no evidence of distant metastases after NAT)	*******

*S* indicates surgery, *IORT* intraoperative radiotherapy, *EBRT* external beam radiotherapy, *CHT* chemotherapy, *NAT* neoadjuvant treatment

*PCD* prospectively collected data, *R* retrospective case-matched study

^a^The Whole study; (+), IORT group; (−), Non-IORT group; *NA* no available; *represented one point, a score of 0 to 9 was assigned to each study and studies achieving a score of 6 or greater were considered high quality

**Table 2:** Characteristics of included studies comparing S + IORT versus S alone

Study	Nation	Study design	Study period	Sample size, n		Mean age (year)		Male, %		Tumor Size (mean, cm)		Median Survival Time (month)		Tumor Staging, n (+) vs. (−)	Study quality
				(+)	(−)	(+)	(−)	(+)	(−)	(+)	(−)	(+)	(−)		
**Hiraoka 1990** [33]	Japan	R	1969–1989	15	19	63.6	61.2	53	73.7	NA	NA	8.8	8.2	Stage I: 0 vs. 2 Stage II: 3 vs. 5 Stage III: 9 vs. 7 Stage IV: 3 vs. 5	*******
**Shibamoto-1 1990** [26]	Japan	R	1975–1989	2	31	60.1^a^	60.9^a^	48.7^a^	67.9^a^	NA	NA	8.5	9.0	Stage I: 0 vs. 11^a^ Stage II: 0 vs. 16^a^ Stage III: 7 vs. 45^a^ Stage IV: 7 vs. 40^a^	*******
**Shibamoto-2 1990** [26]	Japan	R	1975–1989	2	17	60.1^a^	60.9^a^	48.7^a^	67.9^a^	NA	NA	23.0	6.5	Stage I: 0 vs. 11^a^ Stage II: 0 vs. 16^a^ Stage III: 7 vs. 45^a^ Stage IV: 7 vs. 40^a^	*******
**Kawamura 1992** [30]	Japan	R	1978–1990	8	13	67^a^	64^a^	51.4^a^	62.5^a^	NA	NA	18.4	14.3	Stage I: 2 vs. 6^a^ Stage II: 7 vs. 8^a^ Stage III: 28 vs. 26^a^ Stage IV: 0 vs. 0^a^	*******
**Johnstone 1993** [31]	USA	P	1980–1984	7	4	61.5	59.4	25	71.4	NA	NA	NA	NA	Stage I: 0 vs. 4 Stage II-IV: 7 vs. 0	******
**Kasperk 1995** [23]	Germany	R	NA	12	18	62.5^a^	64^a^	69.7^a^	70.7^a^	NA	NA	10.9	12.2	All patients (curative resection)	******
**Dobelbower-2 1997** [14]	USA	R	1980–1995	6	14	58.8	69.1	54.5^a^		4.9	4.7	9.0	6.5	Stage I: 2 vs. 5 Stage II: 2 vs. 5 Stage III: 2 vs. 4	*******
**Ouchi 1998** [27]	Japan	R	1982–1996	5	6	64.7^a^	61.0^a^	66.7^a^	100^a^	NA	NA	8.0	14.0	Stage I: 0 vs. 0 Stage II: 1 vs. 1^a^ Stage III: 5 vs. 6^a^ Stage IV: 0 vs. 0	*******
**Takahashi** **1999** [24]	Japan	R	1985–1997	16	32	NA	NA	NA	NA	NA	NA	10.0	9.0	Stage I: 0 vs. 3 Stage II: 1 vs. 4 Stage III: 5 vs. 14 Stage IVA: 6 vs. 8 Stage IVB: 4 vs. 3	*******
**Koukubo-1 2000** [15]	Japan	R	1980–1997	34	39	63.0^a^	63.0^a^	60.1^a^	60.1^a^	NA	NA	15.0	11.0	All patients (resectable)	*******
**Koukubo-2 2000** [15]	Japan	R	1980–1997	7	14	63.0^a^	63.0^a^	60.1^a^	60.1^a^	NA	NA	8.0	6.0	All patients (resectable)	*******
**Alfieri 2001** [32]	Italy	R	1985–1995	26	20	62.5	58.4	40.0	42.3	3.04	2.73	14.3	10.8	Stage I: 7 vs. 5 Stage II: 5 vs. 5 Stage III: 10 vs. 7 Stage IVA: 4 vs. 3	*******

Shibamoto-1 indicates R0 resection; Shibamoto-2, Non-R0 resection; Koukubo-1, R0 resection; Koukubo-2, Non-R0 resection

*S* indicates surgery, *IORT* intraoperative radiotherapy, *P* prospective study, *R* retrospective case-matched study, *NAT* neoadjuvant treatment, *NA* no available

^a^, The Whole study; (+), IORT group; (−), Non-IORT group; *represented one point, a score of 0 to 9 was assigned to each study and studies achieving a score of 6 or greater were considered high quality

#### Postoperative median survival time

**Fig. 2:**
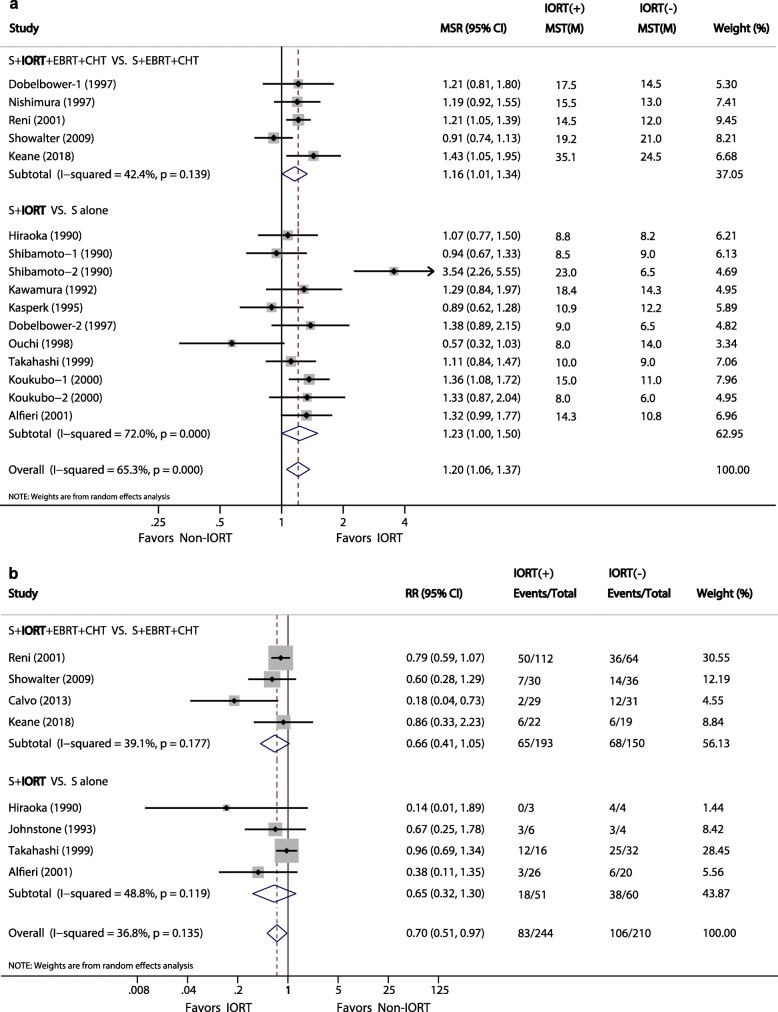
**a**, Forest plots of MSR of median survival time. **b**, Forest plots of RR of local recurrence

The MST was available in 13 studies [10, 14, 15, 23–30, 32, 33]. Overall, patients receiving IORT experienced a remarkably improved MST compared with those who did not receive IORT (MSR, 1.20; 95% CI, 1.06–1.37, *P* = 0.005), with a moderate heterogeneity (*I*^*2*^ = 65.3%). Analyses of the two subgroups showed consistent results that favored the IORT group over the non-IORT group. Patients who underwent S + IORT had a longer MST than those who only had surgery, although the statistical significance was marginal (MSR, 1.23; 95% CI, 1.00–1.50, *P* = 0.05; *I*^*2*^ = 72%), and patients who underwent S + IORT + EBRT + CHT had a significantly longer MST than those who were subjected to S + EBRT + CHT (MSR, 1.16; 95% CI, 1.01–1.34, *P* < 0.05), with low heterogeneity (*I*^*2*^ = 42.4%) (Fig. 2a).

There was evidence of moderate heterogeneity of MSRs across the studies (*I*^*2*^ = 65.3%; *P* < 0.001). Risk estimates barely changed after analyses with fixed effects models, although the substantial heterogeneity remained.

#### Local recurrence

Eight studies [10, 22, 24, 25, 29, 31–33] investigated the association between IORT and postoperative LR. The pooled data showed a significantly reduced risk of LR associated with IORT (RR, 0.7; 95% CI, 0.51–0.97; *P* = 0.03) (Fig. 2b). The results in the two subgroups were consistent, and the heterogeneity of the two subgroups was moderate (*I*^*2*^ = 39.1 and 48.8%, respectively).

#### Postoperative complications and operation-related mortality

**Fig. 3:**
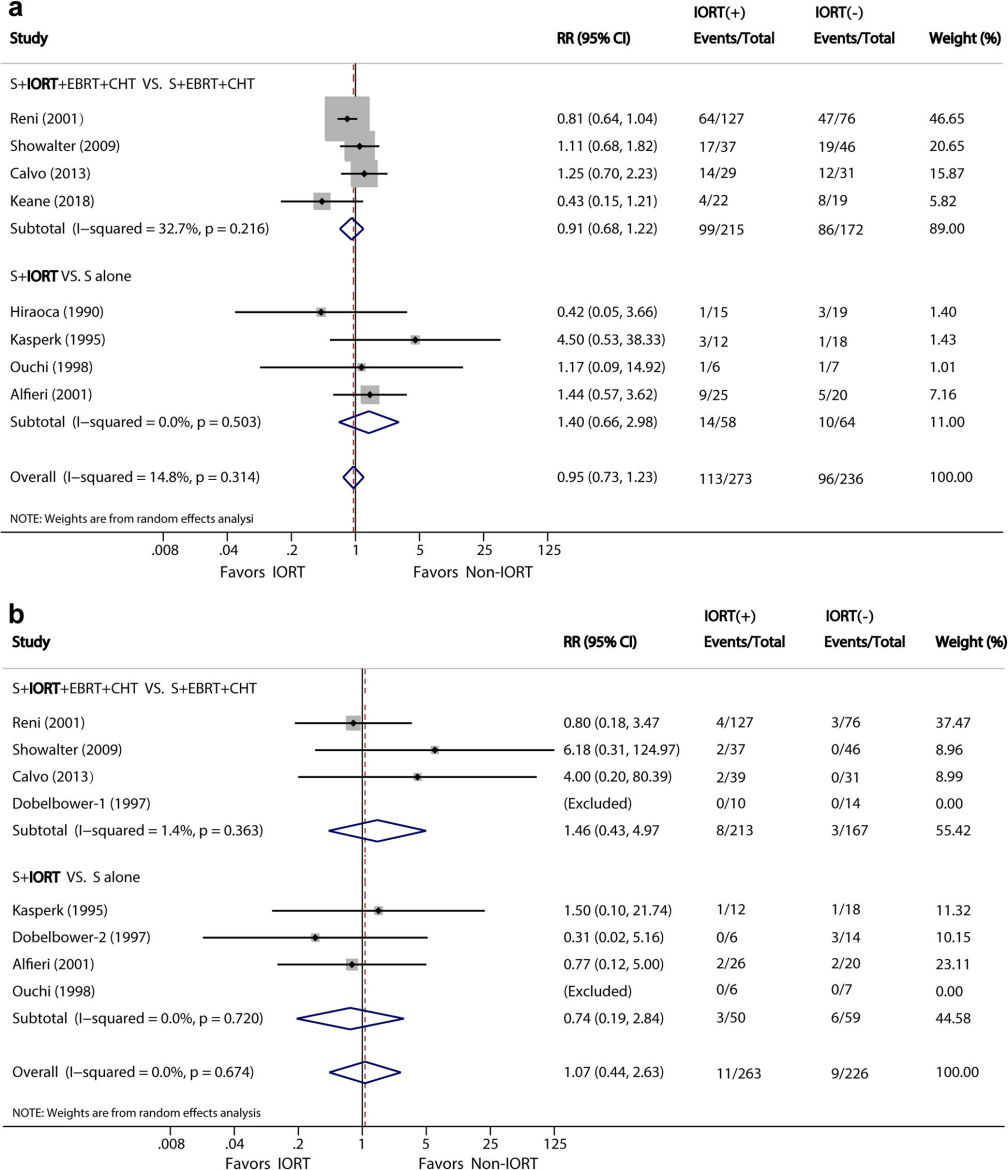
**a**, Forest plots of RR of postoperative complications. **b**, Forest plots of RR of operation-related mortality

The incidences of postoperative complications were reported in 8 studies [10, 22, 23, 25, 27, 32, 33]. The meta-analysis showed no significant difference between the IORT and non-IORT groups (41.4 vs. 40.7%; RR, 0.95; 95% CI, 0.73–1.23, *P* = 0.703) (Fig. 3a). The subgroup analysis showed similar results in the two subgroups (*P* = 0.534 and 0.379, respectively). The heterogeneity of these two subgroups was slight (*I*^*2*^ = 32.7% and 0, respectively). The types of postoperative complications were described in detail in four studies [10, 23, 27, 32] that included 336 patients. Thirty-four (10.1%) of 336 patients had a pancreatic fistula as the most frequent complication. The second and third commonest complications were delayed gastric emptying (7.4%) and abdominal infections (6.5%).

Seven studies [10, 14, 22, 23, 25, 27, 32] reported incidences of overall mortality. The meta-analysis indicated no significant difference in mortality (4.3% vs. 4.0%) between the IORT and non-IORT groups (RR, 1.07; 95% CI, 0.44–2.63) (Fig. 3b). The heterogeneity of these two subgroups was slight (*I*^*2*^ = 1.4% and 0, respectively).

#### Sensitivity analysis

**Fig. 4:**
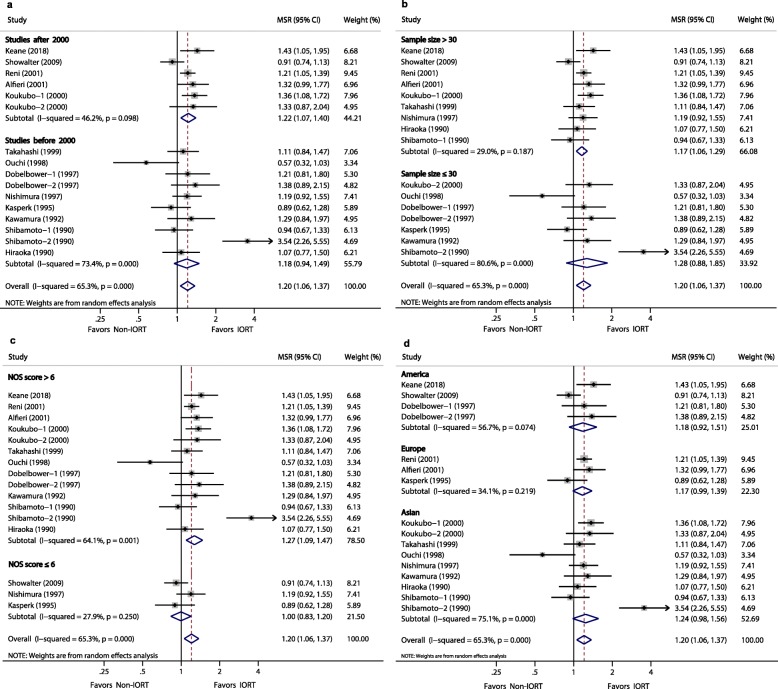
Forest plot of media survival ratio (MSR) for the associations between Media Survival Time (MST) and **a**, publication year; **b**, sample size; **c**, NOS score; **d**, study location

**Fig. 5:**
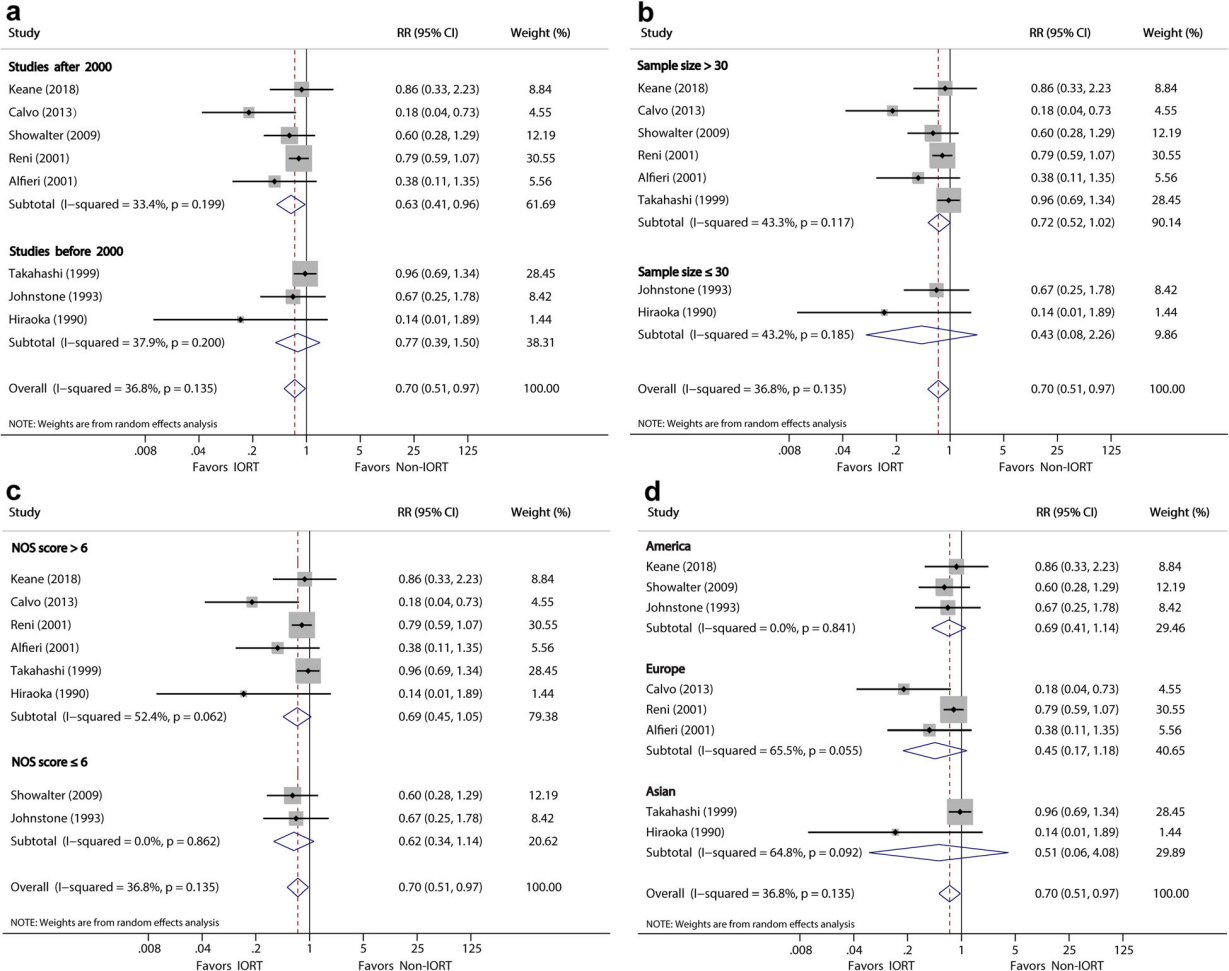
Forest plot of RR for the associations between Local Recurrence (LR) and **a**, publication year; **b**, sample size; **c**, NOS score; **d**, study location

**Fig. 6:**
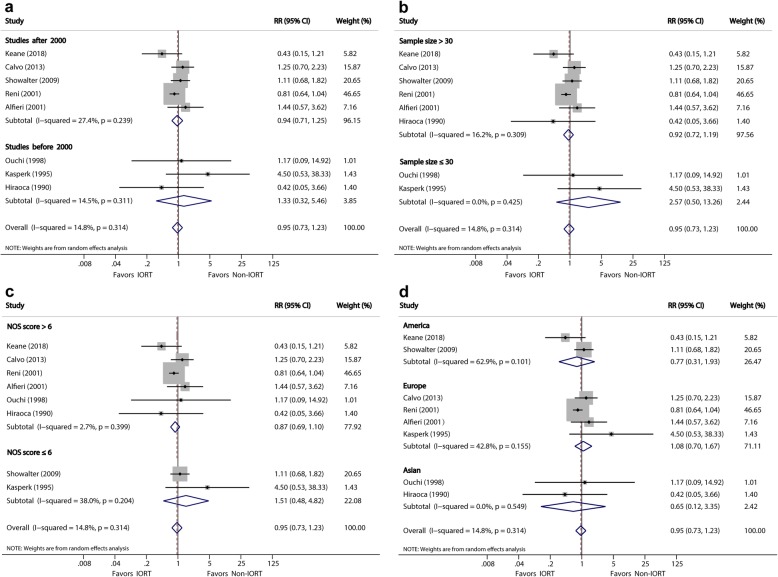
Forest plot of RR for the associations between postoperative complications and **a**, publication year; **b**, sample size; **c**, NOS score; **d**, study location

**Fig. 7:**
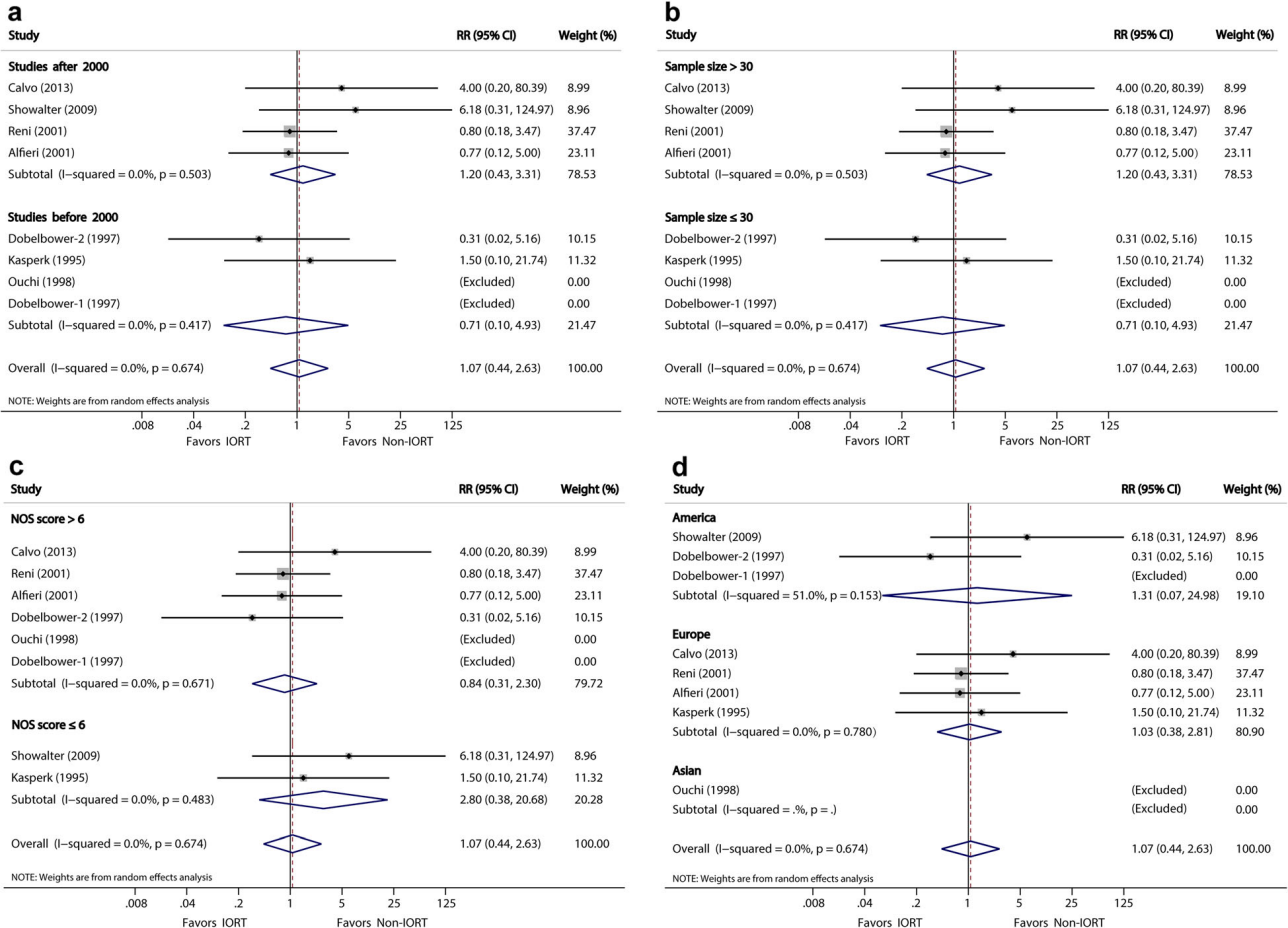
Forest plot of RR for the associations between operation-related mortality and **a**, publication year; **b**, sample size; **c**, NOS score; **d**, study location

Subgroup analyses (Table 3 and Figs. 4, 5, 6 and 7) were performed using the publication year, sample size, study quality, and location in order to explore the potential source of heterogeneity found in these analyses. Subgroup analysis for MST showed a significant difference after year 2000 (MSR, 1.22, 95% CI: 1.07–1.40; *P* = 0.004), sample size >30 (MSR, 1.17, 95% CI: 1.06–1.29; *P* = 0.002), and NOS score >6 (MSR, 1.27, 1.09–1.47; *P* = 0.002). Subgroup analysis for LR showed a significant difference after year 2000 (RR, 0.63; 95% CI, 0.41–0.96; *P* = 0.033).

**Table 3:** Sensitivity Analysis Comparing IORT versus Non-IORT

Stratified analysis	No. Studies	No. Patients	Pooled MSR/RR (95% CI)	***P*** value	Heterogeneity	
					***I***^***2***^ (%)	***P*** value
**(1) Analysis for MST**						
By publication year:						
After 2000y	5	467	1.22 (1.07 to 1.40)	**0.004**	46.2	0.098
Before 2000y	8	296	1.18 (0.94 to 1,49)	0.144	73.4	0.000
By sample size:						
> 30	9	617	1.17 (1.06 to 1.29)	**0.002**	29.0	0.187
≤ 30	6	146	1.28 (0.88 to 1.85)	0.195	80.6	0.000
By NOS score:						
> 6	10	594	1.27 (1.09 to 1.47)	**0.002**	64.1	0.001
≤ 6	3	169	1.00 (0.83 to 1.20)	0.970	27.9	0.250
By study location:						
America	3	168	1.18 (0.92 to 1.51)	0.189	56.7	0.074
Europe	3	279	1.17 (0.99 to 1.39)	0.072	34.1	0.219
Asian	7	316	1.24 (0.98 to 1.56)	0.068	75.1	0.000
**(2) Analysis for LR**						
By publication year:						
After 2000y	5	389	0.63 (0.41 to 0.96)	**0.033**	33.4	0.199
Before 2000y	3	66	0.77 (0.39 to 1.50)	0.439	37.9	0.200
By sample size:						
> 30	6	437	0.72 (0.52 to 1.02)	0.061	43.3	0.117
≤ 30	2	18	0.43 (0.08 to 2.26)	0.316	43.2	0.185
By NOS score:						
> 6	6	312	0.69 (0.45 to 1.05)	0.082	52.4	0.062
≤ 6	2	77	0.62 (0.34 to 1.14)	0.127	0.0	0.862
By study location:						
America	3	118	0.69 (0.41 to 1.14)	0.147	0.0	0.841
Europe	3	282	0.45 (0.17 to 1.18)	0.106	65.5	0.055
Asian	2	55	0.51 (0.06 to 4.08)	0.524	64.8	0.092
**(3) Analysis for complications**						
By publication year:						
After 2000y	5	432	1.33 (0.32 to 5.46)	0.697	14.5	0.311
Before 2000y	3	77	0.94 (0.71 to 1.25)	0.684	27.4	0.239
By sample size:						
> 30	6	466	0.92 (0.72 to 1.19)	0.543	16.2	0.309
≤ 30	2	43	2.57 (0.50 to 13.3)	0.259	0.0	0.425
By NOS score:						
> 6	6	396	0.87 (0.69 to 1.10)	0.240	2.7	0.399
≤ 6	2	113	1.51 (0.48 to 4.82)	0.482	38	0.204
By study location:						
America	2	124	0.77 (0.31 to 1.93)	0.582	62.9	0.101
Europe	4	338	1.08 (0.70 to 1.67)	0.728	42.8	0.155
Asian	2	47	0.65 (0.12 to 3.35)	0.603	0.0	0.549
**(4) Analysis for Mortality**						
By publication year:						
After 2000y	4	392	1.20 (0.43 to 3.31)	0.726	0.0	0.503
Before 2000y	3	87	0.71 (0.10 to 4.93)	0.727	0.0	0.417
By sample size:						
> 30	4	392	1.20 (0.43 to 3.31)	0.503	0.0	0.503
≤ 30	3	86	0.71 (0.10 to 4.93)	0.417	0.0	0.417
By NOS score:						
> 6	5	366	0.84 (0.31 to 2.30)	0.731	0.0	0.671
≤ 6	2	113	2.80 (0.38 to 20.7)	0.312	0.0	0.483
By study location:						
America	2	127	1.31 (0.07 to 25.0)	0.856	51	0.153
Europe	4	339	1.03 (0.38 to 2.81)	0.951	0.0	0.780
Asian	1	13	Not estimable	Not estimable	Not estimable	Not estimable

*MST* indicates median survival time, *LR* local recurrence, *NOS* Newcastle-Ottawa Scale

**Fig. 8:**
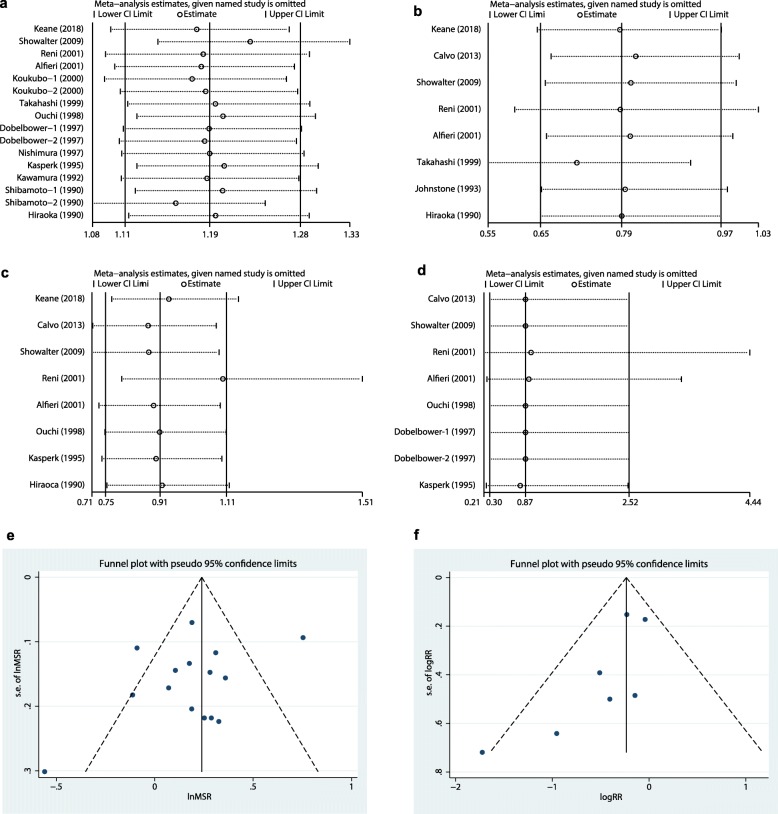
Sensitivity analysis of meta-analysis: **a**, median survival time; **b**, local recurrence; **c**, postoperative complications; **d**, operation-related mortality. Funnel plots for the assessment publication bias: **e**, median survival time; **f**, local recurrence

Sensitivity analysis was performed by sequentially omitting each study. The pooled MSR and 95% CI were not significantly affected by removal of any single study in MST (Fig. 8a). For LR, postoperative complications, and operation-related mortality, the results were similar after the sequential exclusion of each study, which suggested the stability of the meta-analysis (Fig. 8b-d).

#### Publication Bias

Neither funnel plots nor Egger and Begg tests showed evidence of publication bias for the IORT versus non-IORT groups (Egger, *P* = 0.770; Begg, *P* = 0.718) with respect to MST and LR (Fig. 8e-f). After the trim-and-fill method, no additional risk estimate was needed to balance the funnel plot, and the summary effect estimate was not changed.

## Discussion

The prognosis for patients with PC is dismal [35, 36]. In recent years, the concept of multidisciplinary therapy has been proposed for treating PC. As a promising treatment strategy, IORT was introduced as a multimodality management approach to improve both tumor control and overall survival [1]. However, to date, the role of IORT in resectable PC has not been fully understood. To the best of our knowledge, the present study is the first meta-analysis that pooled the results of published research and compared the clinical outcomes in patients with resectable PC who underwent surgery with or without IORT.

LR appears to be the most frequent site of failure and is closely related to the survival rate of PC patients [37]. Our meta-analysis demonstrated that the application of IORT combined with surgery could decrease the LR rate.

Curative resection aiming for margin-negative (R0) status has been advocated to improve overall survival. However, even if a presumed R0 resection is performed, the local tumor recurrence rate remains high in resectable PC patients [38], suggesting that microscopic margin involvement is underestimated [39, 40]. Fortunately, IORT can escalate the radiation dose to the tumor bed and spare adjacent normal tissues from radiation field. High radiobiological effects of IORT could prolong the induction of DNA damage and kill PC stem cell-like cancer cells, leading to the death of residual cancer cells and improvement of locoregional control [41].

The radiation dose that can be safely applied with EBRT is limited due to the tolerance of adjacent structures at risk and potential treatment-related complications. However, IORT has the advantage that it can be used to deliver additional radiation doses to deep-seated cancer residues or risk areas adjacent to radiosensitive critical organs, because these structures can be moved temporarily out of the radiation field.

With respect to overall postoperative survival rate, our meta-analysis indicated that patients who received IORT had a remarkable improvement in MST compared with those who did not receive IORT therapy (*P* = 0.005). In the S + IORT versus surgery alone subgroups, MST was marginally longer in the IORT group than in the non-IORT group (*P* = 0.05). Nevertheless, in the S + IORT+EBRT+CHT versus S + EBRT+CHT subgroups, MST was significantly longer in the IORT group than in the non-IORT group (*P* < 0.05).

The strong ability of PC cells in potentially lethal injury repair (PLDR) and rejoining radiation-induced double-strand breaks (DSBs) results in their intrinsic resistance to radiation [42]. Radiotherapy sensitizers can enhance the radiation damage of DNA and interfere with the cell cycle proliferation [43]. Some studies have confirmed that CHT is a potent radiosensitizer that may promote radiotherapy sensitivity [44, 45]. In addition, as a boosting strategy, IORT combined with EBRT could achieve further dose escalation [46]. Calvo et al. [47] also reported that a combination of 15 Gy IORT boost with 45 Gy EBRT dose used in their study was biologically equivalent to ≥70 Gy EBRT in conventional fractionation. Consequently, the distinct advantage of IORT was in providing further dose escalation when it was used in combination therapy, which was a critical factor to improve local control and overall survival for patients with pancreatic cancer.

Several studies have shown favorable effects of IORT for treatment of patients with pancreatic cancer [10, 29, 48]. However, in the past, the widespread adoption of IORT was hindered due to limitations of beam energy, dose rate, and equipment availability [49]. Currently, the associated postoperative complications such as pancreatic fistula, delayed gastric emptying, and abdominal infections are additional factors that may affect clinical outcomes for PC patients [50]. Nonetheless, our meta-analysis showed that IORT did not cause an increase in postoperative complications and operation-related mortality, which occurred in 41.4% vs. 40.7 and 4.3% vs. 4.0% in both groups, respectively. IORT allows precise application of high radiation dose to the planning target volume (PTV) with minimal exposure of adjacent tissues, such as small intestine, liver, and kidney, to exorbitant radiotherapy dose. Therefore, IORT could be safely delivered to affected tissues in resectable PC during surgical resection. Although the current study does not provide a specific plan of combination therapy, our findings suggest that IORT should be considered as a potential component of an adjuvant multiple-treatment strategy. With this information, adjuvant systemic therapies for resectable PC should be further improved.

This meta-analysis had several limitations. First, most of the included trials had small sample sizes. Nevertheless, analysis of the pooled data clearly showed a superior effect of surgery combined with IORT with regards to MST and LR when compared to non-IORT groups. As postoperative complications and operation-related mortality rates were similar between the two groups, applying IORT did not cause additional risks or side effects to the patients.

Second, there was substantial heterogeneity in our meta-analysis. In fact, owing to potential confounders, such as population characteristics, year of publication, the study sample sizes, etc., we conducted subgroup and sensitivity analyses to identify the source of heterogeneity and confirm the stability of our findings. Risk estimates barely changed after analyses with fixed effects models; however, substantial heterogeneity remained. When the analysis only included studies from selected periods when the publications were produced (before or after 2000), the overall polled data showed a significant difference that favored combined IORT (MSR, 1.20, 95% CI: 1.06–1.37; *P* = 0.005), especially for studies conducted after 2000 (MSR, 1.22, 95% CI: 1.07–1.40; *P* = 0.004), which appears to be attributable to combination therapy.

Using sensitivity analysis, the main source of heterogeneity was identified from the study by Shibamoto et al., wherein the imbalance of sample sizes between the IORT and non-IORT groups was significant (*n* = 4 and 48, respectively). After excluding this single study, the heterogeneity reduced from moderate (*I*^*2*^ = 65.3%; *P* = 0.000) to low level (*I*^*2*^ = 30.6%; *P* = 0.132), and the pooled estimate still reached statistical significance (MSR = 1.17; 95% CI: 1.06–1.28, *P* = 0.002).

Third, most of the included studies in the present meta-analysis were retrospective analyses; thus, further large randomized controlled trials are warranted to confirm these findings.

## Conclusions

In summary, this meta-analysis indicated that IORT significantly improved the locoregional control and overall survival for patients with resectable PC without increasing postoperative complications and operation-related mortality rate. Therefore, IORT is a safe and effective procedure that is associated with improved long-term clinical outcomes for patients with resectable PC. In addition, these findings demonstrated the importance of combination therapy.

## Supplementary information

**Additional file 1.**

## Data Availability

All data generated or analysed during this study are included in this published article [and its supplementary information files].

## Abbreviations

IORT: Intraoperative radiotherapy
PC: Pancreatic cancer
MST: Median survival time
LR: Local recurrence
MSR: Median survival rate
CI: Confidence interval
RR: Relative risk
CHT: Chemotherapy
EBRT: External beam radiotherapy
S: Surgery
PLDR: Potentially lethal injury repair
DSBs: Double-strand breaks
PTV: Planning target volume
PRISMA: Preferred reporting items for systematic reviews and meta-analysis
M: Month
