# Supplementary material for: The role of intraoperative radiation therapy in resectable pancreatic cancer: a systematic review and meta-analysis
Source: Radiat Oncol. 2020 Apr 9;15:76. doi: 10.1186/s13014-020-01511-9 (PMC7147036; doi:10.1186/s13014-020-01511-9)
Supplement: Supplementary file 1 — Additional file 1. [file 13014_2020_1511_MOESM1_ESM.zip › Table 4.docx]

| Table 4. Relevant supplementary information (S+IORT versus S alone). | | | | | | |
| --- | --- | --- | --- | --- | --- | --- |
| **Study** | **IORT** | **Surgical procedures, n**  **（IORT vs. Non-IORT）** | **Complications, n**  **（****IORT vs. Non-IORT）** | **Tumor Site, n**  **（IORT vs. Non-IORT）** | | |
|  |  |  |  | Head | Body | Tail |
| **Hiraoka**  **1990** | 1.**Equipment:** NA;  2.**Diameter:** using a 6-8cm circular applicator;  3. **Range:** the operative field, including the coeliac axis and superior mesenteric artery. The pancreatic remnant and the bile duct were kept outside of the irradiation field (Hiraoka-1984-Fig. 1);  4.**Radiation time:** After resection of the pancreatic lesion;  **5. Dose:** a dose of 30 Gy of 8MeV electrons from a linear accelerator . | All patients had a standard resection defined as a pancreatectomy with resection of the local and secondary group of lymph nodes draining the pancreas. | 1.Anastomotic leakage: 5 patients*;  2.Intestinal bleeding: 1 patient*;  3. Three of these patients were from Non-IORT Group; one from IORT Group. | NA | | |
| **Shibamoto**  **1990** | 1.**Equipment:** NA;  2.**Diameter:** a 5.8 cmX3.6 cm field;  3.**Range:** a single dose of 2 500 R 18 MeV electron radiation was delivered to the surface of the main mass;  4.**Radiation time:** After choledochojejunostomy;  **5. Dose:** The IORT dose was 25 Gy for curative resection and 30-33 Gy for non-curative or no resection. | 1.Total pancreatectomy*: 20;  2.Pancreaticoduodenectomy*: 62;  3.Distal pancreatectomy*: 18;  4.Bypass*: 70;  5. Laparotomy*: 21. | NA | 135* | 67* | NA |
| **Kawamura**  **1992** | 1.**Equipment:** NA;  2.**Diameter:** Circular cones with an inside diameter of 6 to 10 cm;  3.**Range:** the residual tumor or tumor bed;  4.**Radiation time:** NA;  **5. Dose:** the energy of the electron beam was 8 MeV. | 1.Total pancreatectomy: 4 vs. 9*;  2.Pancreaticoduodenectomy: 9 vs. 12*;  3.Distal pancreatectomy: 3 vs. 1*;  4. Gastrectomy: 31 vs. 22*. | NA | 16 vs. 22* | 5 vs. 6* | 0 vs. 1* |
| **Johnstone**  **1993** | 1.**Equipment:** NA;  **2.Diameter:** NA;  3.**Range:** Porta hepatis; pancreatic bed; celiac axis; RUQ; iliac access; biliary tree; SMA; LUQ; porta hepatis; mesenteric root; Pancreatic bed; portal vein;  4.**Radiation time:** NA;  5. **Dose:** 20 Gy IORT using 9- 12 MeV electrons. | 1.Total pancreatectomy: 4 vs. 3;  2.Pancreaticoduodenectomy: 0 vs. 1;  3. Radical pancreatectomy: 3 vs. 0. | There were seven perioperative deaths (27%). Two patients who died of sepsis within 2 months of surgery, which were felt to be treatment-related complications. In total, 4 patients died of infectious. Causes , and 3 of vascular complications. | NA | | |
| **Kasperk**  **1995** | 1.**Equipment:** NA;  2.**Diameter:** Circular cones with an inside diameter of 6 to 10 cm;  3.**Range:** after exposure of the tumor or after resection and clearance of the tumor bed, a transparent acrylic cylinder of adequate size is placed in the abdomen, keeping adjacent organs out of the radiation field;  4.**Radiation time:** NA;  **5. Dose:** range from 9 to 20 MeV and 10 to 20 Gy. | All patients recieved R0 resection.  R0 resection: 12 vs. 18. | 1.superficial wound infections: 12% vs. 12%*;  2.anastomotic leakage:2 vs. 0*;  3.peripancreatic abscess formation:2 vs. 0*, 4.pancreatitis in the pancreatic remnant:1 vs. 0*;  5.pancreatic fistula:2 vs. 1*;  6.intra-abdominal haemorrhage:0 vs. 1*;  7. Ileus: 0 vs. 1*. | 57* | 10* | 6* |
| **Dobelbower-2**  **1997** | 1.**Equipment:**NA;  2.**Diameter**:ranged from 1.7 to 4 inches in internal diameter;  3.**Range:**Tumor bed irradiation was conducted with three, four, or more custom-shaped fields at 1.8-2.0 Gy/ fraction, five fractions weekly;  4.**Radiation time:** NA;  **5. Dose:** ranged from 10 to 20 Gy (modal dose 15). | All patients with radical surgery.  1.PPPD^#^ : 1 vs. 0;  2.pancreatectomy: 2 vs. 0;  3. Whipple procedure: 11 vs. 6. | **Early complications:**  1.Gastrointestinal symptoms: 3 vs. 4;  2.Drain or wound infection: 1 vs. 1;  3.Fever: 1 vs. 1;  4.Respiratory (pneumonia, pleural effusion, atelectasis ): 2 vs. 2;  5.Small bowel/gastric outlet obstruction: 2 vs. 2;  6.Septicemia: 1 vs. 1;  7.Fistula: 1 vs. 0;  8.Abdominal abscess: 1 vs. 0;  9. Acute renal failure: 0 vs. 1. | 6 vs. 14 | NA | |
| **Ouchi**  **1998** | 1.**Equipment:** Shimazu BT-20, Kyoto, Japan;  2.**Diameter:** ranging from 4 to 12 cm were used; an 8-cm cone was used in about 50% of all irradiated patients;  3.**Range:** after exposure of the tumor or after resection and clearance of the tumor bed, a transparent acrylic cylinder of adequate size is placed in the abdomen, keeping adjacent organs out of the radiation field;  4.**Radiation time:** Treatment was delivered before reconstruction;  **5. Dose:** The IORT dose was 20 to 25 Gy for pancreatic resection and 25 to 30 Gy for surgical bypass (a 78-year-old woman undergoing PD followed by IORT at a radiation dose of 15 Gy. | Pancreaticoduodenectomy (PD): 6 vs. 7*. | Anastomotic leak: 1 vs 1. | NA | | |
| **Takahashi**  **1999** | 1.**Equipment:** NA;  2.**Diameter:** NA;  3.**Range:** retroperitoneum;  4.**Radiation time:** after resection of the pancreatic head before reconstruction;  **5. Dose:** administer 20–30 Gy at 9– 12 MeV. | 1.PD: 9 vs. 18;  2.PPPD: 2 vs. 7;  3. TP: 5 vs. 7. | **PPPD (n=29) VS. PD (n=62)**  1.Intra-abdominal hemorrhage pancreatic: 3 vs. 2;  2.juice leakage: 3 vs. 1;  3.Gastrointestinal hemorrhage: 2 vs. 1;  4.Delayed gastric emptying: 1 vs. 1;  5.Hepatic abscess: 1 vs. 0;  6.Bile leakage: 0 vs. 2;  7. Ileus: 0 vs. 1. | NA | | |
| **Koukubo**  **2000** | 1.**Equipment:** NA;  2.**Diameter:** Treatment cones of 5×5 to7×7 cm square, 5- to 7-cm diameter circle, or pentagon were frequently used;  3.**Range:** covered the tumor bed and para-aortic lymph node area;  4.**Radiation time:** NA;  **5. Dose:** The **IORT** dose was 20–30 Gy with median dose of 25 Gy in a single fraction. | All patients had undergone total or regional pancreatectomy with radical lymph node dissection of the para-aortic area as well as peripancreatic region, and dissection of nerve plexus along the superior mesenteric artery, celiac arterial axis, and aorta. | NA | NA | | |
| **Alfieri**  **2001** | 1.**Equipment:** NA;  2.**Diameter:** NA;  **3.Range:** The tumor bed, including the portal vein, the splenomesenteric confluence, the superior mesenteric vein up to its branches, the celiac trunk, the hepatic artery up to its division, the proximal splenic vessel, the suprarenal aorta, and the caval vein;  4.**Radiation time:** after resection;  **5. Dose:** A dose of 10Gy was delivered with a 6-MeF electron beam. | **Extent of pancreatectomy:**  Partial: 25 vs. 13;  Total: 1 vs. 7.  **Reconstruction:**  Whipple:1 vs. 6;  traverso-longmire: 25 vs. 14.  **Pancreatic stump anastomosis:**  Pancreatojejunostomy: 24 vs. 19  Wirsung jejunostomy: 2 vs. 0  No anastomosis: 0 vs. 1 | 1.Reoperation: 5 vs. 2;  2.Bleeding: 2 vs. 1;  3.Pancreatojejunostomy leakage: 0 vs. 1;  4.intra-abdominal abscess: 0 vs. 1;  5. Fascial dehiscence: 2 vs. 0. | 26 vs. 20 | NA | |
| PD indicates pancreatoduodenectomy; TP, total pancreatectomy; PPPD, pylorus-preserving pancreatoduodenectom; S, surgery; IORT, intraoperative radiotherapy; EBRT, external beam radiotherapy; CHT, chemotherapy.  *, The Whole study; NA, no [available](C:/Users/lenovo/AppData/Local/Youdao/Dict/Application/6.3.69.8341/resultui/frame/javascript:void(0);). | | | | | | |
