# Supplementary material for: The role of intraoperative radiation therapy in resectable pancreatic cancer: a systematic review and meta-analysis
Source: Radiat Oncol. 2020 Apr 9;15:76. doi: 10.1186/s13014-020-01511-9 (PMC7147036; doi:10.1186/s13014-020-01511-9)
Supplement: Supplementary file 1 — Additional file 1. [file 13014_2020_1511_MOESM1_ESM.zip › Table 5.docx]

| Table 5. Relevant supplementary information (S+IORT+EBRT+CHT versus S+EBRT+CHT). | | | | | | | | |
| --- | --- | --- | --- | --- | --- | --- | --- | --- |
| **Study** | **IORT** | **EBRT** | **CHT regimens, n**  **（IORT vs. Non-IORT）** | **Surgical procedures, n**  **（IORT vs. Non-IORT）** | **Complications, n**  **（IORT vs. Non-IORT）** | **Tumor Site, n**  **（IORT vs. Non-IORT）** | | |
|  |  |  |  |  |  | Head | Body | Tail |
| **Dobelbower-1**  **1997** | 1.**Equipment**: NA;  2. **Diameter:** ranged from 1.7 to 4 inches in internal diameter.  3.**Range:** Tumor bed irradiation was conducted with three, four, or more custom-shaped fields at 1.8-2.0 Gy / fraction, five fractions weekly;  4.**Radiation time:** NA;  **5. Dose:** ranged from 10 to 25 Gy (modal dose 15). | **Dose:** doses ranging from 50 to 67.7 Gy (modal dose 50.4). | Five fluorouracil (5-FU):  9 vs. 9. | **In Non-IORT group:** All 14 patients with radical surgery;  **In IORT group:**  1.Distal pancreatectomy:3 patients;  2.Pancreatectomy: 3 patients;  3. Whipple resection: 4 patients. | **Early complications:**  1.Gastrointestinal symptoms:8 vs. 6;  2.Drain or wound infection:1 vs. 2;  3.Fever:1 vs. 2;  4.Respiratory (pneumonia, pleural effusion, atelectasis):0 vs. 1;  5.Small bowel/gastric outlet obstruction:0 vs. 1;  6.Fistula:0 vs. 1;  7.Persistent jaundice:0 vs. 2;  8. Ascites: 3 vs. 0. | 7 vs. 14 | 2 vs. 0 | 2 vs. 0 |
| **Nishi**  **1997** | 1.**Equipment:** NA;  2.**Diameter:** 5-7 cm in diameter were most frequently used;  3.**Range:** The IORT field covered the tumor bed including the origin of the celiac trunk and the superior mesenteric artery for resected pancreatic cancer, the gastrointestinal tract was not included in the IORT field;  4.**Radiation time:** NA;  **5. Dose:** 8-12 MeV for resected pancreatic cancer, IORT dose was 20-25 Gy for curative, and 30-33 Gy for noncurative resection. | 1**. Direction:** EBRT mostly use three portals from the anterior, left, and right directions.  2. **Diameter:** The average field sizes were 10 × 10cm from the anterior direction and 10 x 7cm from the lateral directions.  3. **Dose:** The usual EBRT dose was approximately 50 Gy for curative resection. | Various combination of 1.mitomycin C (2-8 mg);  2.epirubicin(10-30mg);  3.5-FU; 250-500 mg);  or cisplatin ( 10-50 mg);  were injected every 2-4 weeks during and after postoperative EBRT for 6-12 months or until death. | 1.Total pancreatectomy:20*;  2.Distal pancreatectomy:32*;  3. Pancreaticoduodenectomy:105*. | Complications associated with IORT:  1.Gastrointestinal ulcer:11;  2.Intestinal perforation:2:  3.Arterial rupture, aneurysm, and occlusion:4:  4.Abdominal abscess:3;  5.Hepatic abscess:3;  6.DIC:3;  7. Ileus: 2. | 36 vs. 26* | 4 vs. 12* | |
| **Reni**  **2001** | 1.**Equipment:** Toshiba LMR15 linear accelerator (Nasu, Tokyo, Japan) until December 1987 and, thereafter, with a Varian Clinac accelerator (Varian, Palo Alto, CA);  2.**Diameter:** an acrylic cylinder of 4- to 12-cm diameter and with an inclination angle of 0–30°;  3.**Range:** the tumor bed with appropriate margins(celiac axis, the superior mesenteric artery, and the edge of the pancreatic stump)  4.**Radiation time:** irradiation field after pancreaticoduodenectomy;  **5. Dose:** 10–25 Gy (median, 17.5 Gy) were delivered with electron beam energies of 6–12 MeV. | 1.**Direction:** NA;  2.**Diameter:** EBRT to the tumor bed plus 3- to 5-cm margins;  3. **Dose:** was delivered with photon beam energies of 6–18 MV in 56 (28%) patients with a median dose of 43.2 Gy (range, 1.8–59.4 Gy). | Eight postoperative chemotherapy regimens were utilized in 82 (40%) cases:  **1.**5-FU+anthracyclines±mitomycin in 46% of cases;  **2.**5-FU, epirubicin and cisplatin in 36% of cases; **3.**PEF-G regimen (cisplatin, epirubicin, 5-fluorouracil, and gemcitabine) in 11% of cases; **4.**gemcitabine±5-fluorouracil±cisplatin in 7% of cases. | 1.Pancreaticoduodenectomy:  17 vs. 65 ;  2.Distal pancreatectomy：  21 vs 9；  3.Total pancreatectomy：  2 vs 2. | 1.Pancreatic fistula: 17 vs 14;  2.Delayed gastric emptying: 15 vs 10;  3.Abdominal infectious: 12 vs 10;  4.Intraperitoneal bleeding: 8 vs 5;  5. Other complications (Intraperitoneal or wound infection): 12 vs 8. | 107 vs. 66 | NA | |
| **Showalter**  **2009** | 1.**Equipment:** NA;  2.**Diameter:** NA;  3. **Range:** encompassing the pancreatic tumor bed within the 90% isodose line. Regional lymph nodes were not included in the target volume for most cases;  4.**Radiation time:** At the time of surgery, not explain whether before digestive anastomosis or not;  **5. Dose:** IORT was delivered using 6–15 MeV electrons and cone sizes selected in order to deliver a dose of 10–20 Gy . | Adjuvant EBRT was also delivered, a dose of 45–50.4 Gy was prescribed using a conformal, four-field radiation technique. | the current study does not explain the detailed plan of CHT. | All patients treated with Pancreatoduodenectomy. | The current study does not explain the type of complications in detail, just reported the number of complilcations. | NA | | |
| **Calvo**  **2013** | 1.**Equipment:** NA;  2.**Diameter:** Beveled (15e30°) circular applicators (size range, 7-10 cm);  3.**Range:** comprising the retroperitoneum, vascular structures, and tumor bed extending from the transected bile duct superiorly to the right kidney laterally and to the pancreatic remnant medially), using a median energy of 10 MeV (range, 9-18 MeV. The bile duct and pancreatic remnant were excluded from the treatment field;  4.**Radiation time:** After tumor resection and before digestive anastomosis;  **5.Dose:** 10-15 Gy (median, 15 Gy) was delivered in a single fraction to a 1-field PTV (comprising the retroperitoneum, vascular structures, and tumor bed extending from the transected bile duct superiorly to the right kidney laterally and to the pancreatic remnant medially), using a median energy of 10 MeV (range, 9e 18 MeV). | 1.**Direction:** using the 3D conformal field technique;  2.**Diameter:** Clinical target volume was set to cover the tumor bed (with a≥2-cm margin) and primary lymph nodes);  3. **Dose:** A total median dose of 45 Gy (range, 45-50.4 Gy [1.8 Gy/5d/wk]) was prescribed to the isodose line that covered the PTV to obtain a homogeneity±5% of the prescribed dose. | Oral Tegafur was administered concomitantly to the patients at 1,200 mg/d (400 mg/8 h) throughout the total duration of the radiationtherapy, including weekends and holidays or operative inter-ruptions in which no radiation treatment was delivered. | 1.Duodeno-cephalo-pancreatectomy： 24 vs. 24;  2.Total pancreatectomy：  4 vs. 5;  3.Distal pancreatectomy：  1 vs. 2. | 1.Gastrointestinal: 6*;  2.Soft tissue abscess: 1*;  3.Wound infection: 6*;  4. Peripheral neuropathy: 3*.  5.Cardiac:4*;  6. Pulmonary: 6*. | 26 vs. 28 | 1 vs. 2 | 2 vs. 1 |
| **Keane**  **2018** | IORT was administered in patients who were able to undergo resection after neoadjuvant treatment.  1.**Equipment:** NA;  2.**Diameter:** a metal applicator (median diameter 5 cm; range, 4 to 8 cm) was selected and used to enclose either the pancreatic tumor or resection bed with an approximately 1 cm surrounding margin;  3.**Range:** to the tumor and regional lymph nodes;  **4.Radiation time:** Accompanying procedures were often performed before surgical closure: gastrojejunostomy or gastroenterostomy; hepaticojejunostomy; cholecystectomy; and/or choledochojejunostomy, choledochoduodenostomy, or cholecystojejunostomy;  5. Dose: After resection, a median dose of 10 Gy (range, 8 to 13 Gy) was delivered to the resection bed and positive margins. | 1.**Direction:** NA;  2. **Diameter:** The clinical target volume included these areas as well as the porta hepatis, celiac axis, SMA, and pancreaticoduodenal nodes. The planning target volume was typically a 0.5-cm radial and 0.7-cm craniocaudal expansion on the clinical target volume;  3. **Dose:** Median EBRT dose was 50.4 Gy (range, 24 to 55 Gy). | All patients received intensive NAT.  1. FOLFIRINOX (n = 59)*;  2.gemcitabine with nab-paclitaxel (n = 4) *;  3. Patients who were started on FOLFOX with the intent to intensify to FOLFIRINOX if possible (n = 5)*. | 1.Whipple:16 vs. 14;  2. Distal pancreatectomy:6 vs. 5. | The current study does not explain the type of complications in detail, just reported the number of complications. | NA | | |
| S indicates surgery; IORT intraoperative radiotherapy; EBRT external beam radiotherapy; CHT chemotherapy.  *, The Whole study; NA, no [available](C:/Users/lenovo/AppData/Local/Youdao/Dict/Application/6.3.69.8341/resultui/frame/javascript:void(0);). | | | | | | | | |

.
