# Supplementary material for: The role of intraoperative radiation therapy in resectable pancreatic cancer: a systematic review and meta-analysis
Source: Radiat Oncol. 2020 Apr 9;15:76. doi: 10.1186/s13014-020-01511-9 (PMC7147036; doi:10.1186/s13014-020-01511-9)
Supplement: Supplementary file 1 — Additional file 1. [file 13014_2020_1511_MOESM1_ESM.zip › Supplementary Material.doc]

**Supplementary Material**

**The Role of Intraoperative Radiation Therapy in Resectable Pancreatic Cancer: A Systematic Review and Meta-analysis**

**File 1.** Search Strategy.

**Table 3.** Relevant supplementary information (S+IORT+EBRT+CHT versus S+EBRT+CHT).

**Table 4.** Relevant supplementary information (S+IORT versus S alone).

**File 1.** Search Strategy.

**Pubmed: 408**

**Keywords for pancreatic cancer	13040**

(((((Pancreatic malignant tumor[Title/Abstract]) OR Pancreatic adenocarcinoma[Title/Abstract]) OR Pancreatic carcinoma[Title/Abstract])) OR ((((((((((((((((((((Neoplasm, Pancreatic[Title/Abstract]) OR Pancreatic Neoplasm[Title/Abstract]) OR pancreas Neoplasms[Title/Abstract]) OR Neoplasm, Pancreas[Title/Abstract]) OR Neoplasms, Pancreas[Title/Abstract]) OR Pancreas Neoplasm Neoplasms, Pancreatic[Title/Abstract]) OR Cancer of Pancreas[Title/Abstract]) OR Pancreas Cancers[Title/Abstract]) OR Pancreas Cancer[Title/Abstract]) OR Cancer, Pancreas[Title/Abstract]) OR Cancers, Pancreas[Title/Abstract]) OR Pancreatic Cancer[Title/Abstract]) OR Cancer, Pancreatic[Title/Abstract]) OR Cancers, Pancreatic[Title/Abstract]) OR Pancreatic Cancers[Title/Abstract]) OR Cancer of the Pancreas[Title/Abstract])) OR "Pancreatic Neoplasms"[Mesh]))))

**Keywords for intraoperative Radiation Therapy	4790**

((((((Intraoperative[Title/Abstract]) OR intra-operative[Title/Abstract])) AND ((((((((((((((((((((((Radiotherapies[Title/Abstract]) OR Radiation Therapy[Title/Abstract]) OR Radiation Therapies[Title/Abstract]) OR Therapies, Radiation[Title/Abstract]) OR Therapy, Radiation[Title/Abstract]) OR Radiation Treatment[Title/Abstract]) OR Radiation Treatments[Title/Abstract]) OR Treatment, Radiation[Title/Abstract]) OR Radiotherapy, Targeted[Title/Abstract]) OR Radiotherapies, Targeted[Title/Abstract]) OR Targeted Radiotherapies[Title/Abstract]) OR Targeted Radiotherapy[Title/Abstract]) OR Targeted Radiation Therapy[Title/Abstract]) OR Targeted Radiation Therapies[Title/Abstract]) OR Radiation Therapies, Targeted[Title/Abstract]) OR Therapies, Targeted Radiation[Title/Abstract]) OR Therapy, Targeted Radiation[Title/Abstract]) OR Radiation Therapy, Targeted[Title/Abstract])) OR "Radiotherapy"[Mesh]))))) OR IORT[Title/Abstract])

**Intraoperative radiotherapy for pancreatic cancer	408**

((((((((((Intraoperative[Title/Abstract]) OR intra-operative[Title/Abstract])) AND ((((((((((((((((((((((Radiotherapies[Title/Abstract]) OR Radiation Therapy[Title/Abstract]) OR Radiation Therapies[Title/Abstract]) OR Therapies, Radiation[Title/Abstract]) OR Therapy, Radiation[Title/Abstract]) OR Radiation Treatment[Title/Abstract]) OR Radiation Treatments[Title/Abstract]) OR Treatment, Radiation[Title/Abstract]) OR Radiotherapy, Targeted[Title/Abstract]) OR Radiotherapies, Targeted[Title/Abstract]) OR Targeted Radiotherapies[Title/Abstract]) OR Targeted Radiotherapy[Title/Abstract]) OR Targeted Radiation Therapy[Title/Abstract]) OR Targeted Radiation Therapies[Title/Abstract]) OR Radiation Therapies, Targeted[Title/Abstract]) OR Therapies, Targeted Radiation[Title/Abstract]) OR Therapy, Targeted Radiation[Title/Abstract]) OR Radiation Therapy, Targeted[Title/Abstract])) OR "Radiotherapy"[Mesh]))))) OR IORT[Title/Abstract])))) AND (((((Pancreatic malignant tumor[Title/Abstract]) OR Pancreatic adenocarcinoma[Title/Abstract]) OR Pancreatic carcinoma[Title/Abstract])) OR ((((((((((((((((((((Neoplasm, Pancreatic[Title/Abstract]) OR Pancreatic Neoplasm[Title/Abstract]) OR pancreas Neoplasms[Title/Abstract]) OR Neoplasm, Pancreas[Title/Abstract]) OR Neoplasms, Pancreas[Title/Abstract]) OR Pancreas Neoplasm Neoplasms, Pancreatic[Title/Abstract]) OR Cancer of Pancreas[Title/Abstract]) OR Pancreas Cancers[Title/Abstract]) OR Pancreas Cancer[Title/Abstract]) OR Cancer, Pancreas[Title/Abstract]) OR Cancers, Pancreas[Title/Abstract]) OR Pancreatic Cancer[Title/Abstract]) OR Cancer, Pancreatic[Title/Abstract]) OR Cancers, Pancreatic[Title/Abstract]) OR Pancreatic Cancers[Title/Abstract]) OR Cancer of the Pancreas[Title/Abstract])) OR "Pancreatic Neoplasms"[Mesh])))))

**Embase: 662**

**Keywords for pancreatic cancer	104683**

#2	'pancreas cancer'/exp	92063

#3	'neoplasm, pancreatic':ab,ti	19

#4	'pancreatic neoplasm':ab,ti	791

#5	'pancreatic neoplasms':ab,ti	1503

#6	'pancreas neoplasms':ab,ti	28

#7	'neoplasm, pancreas':ab,ti	5

#8	'neoplasms, pancreas':ab,ti	2

#9	'pancreas neoplasm':ab,ti	18

#10	'neoplasms, pancreatic':ab,ti	27

#11	'cancer of pancreas':ab,ti	90

#12	'pancreas cancers':ab,ti	170

#13	'pancreas cancer':ab,ti	2058

#14	'pancreatic cancer':ab,ti	52750

#15	'pancreatic cancers':ab,ti	3940

#16	'cancer, pancreas':ab,ti	83

#17	'cancers, pancreas':ab,ti	18

#18	'cancer, pancreatic':ab,ti	786

#19	'cancers, pancreatic':ab,ti	104

#20	'cancer of the pancreas':ab,ti	1234

#21	'pancreatic carcinoma':ab,ti	8303

#22	'pancreas adenocarcinoma':ab,ti	252

#23	'pancreatic adenocarcinoma':ab,ti	10541

#24	'pancreatic malignant tumor':ab,ti	29

#25	'pancreas carcinoma':ab,ti	497

#26	#2 OR #3 OR #4 OR #5 OR #6 OR #7 OR #8 OR #9 OR #10 OR #11 OR #12 OR #13 OR #14 OR #15 OR #16 OR #17 OR #18 OR #19 OR #20 OR #21 OR #22 OR #23 OR #24 OR #25	104683

**Keywords for radiotherapy	536563**

#27	'radiotherapy'/exp	508349

#28	'radiotherapies':ab,ti	426

#29	'radiation therapy':ab,ti	107697

#30	'radiation therapies':ab,ti	480

#31	'therapies, radiation':ab,ti	62

#32	'therapy, radiation':ab,ti	696

#33	'radiation treatment':ab,ti	13210

#34	'radiation treatments':ab,ti	1262

#35	'treatment, radiation':ab,ti	545

#36	'radiotherapy, targeted':ab,ti	70

#37	'radiotherapies, targeted':ab,ti	2

#38	'targeted radiotherapies':ab,ti	8

#39	'targeted radiotherapy':ab,ti	762

#40	'radiation therapies, targeted':ab,ti	0

#41	'radiation therapy, targeted':ab,ti	22

#42	'targeted radiation therapies':ab,ti	5

#43	'targeted radiation therapy':ab,ti	138

#44	'therapies, targeted radiation':ab,ti	0

#45	'therapy, targeted radiation':ab,ti	1

#46	#27 OR #28 OR #29 OR #30 OR #31 OR #32 OR #33 OR #34 OR #35 OR #36 OR #37 OR #38 OR #39 OR #40 OR #41 OR #42 OR #43 OR #44 OR #45	536563

**Keywords for intraoperative	280142**

#47	'intraoperative period'/exp	171837

#48	'intraoperative':ab,ti	148690

#49	'intra-operative':ab,ti	21531

#50	#47 OR #48 OR #49	280142

**intraoperative radiotherapy	10573**

#51	#46 AND #50	10313

#53	'iort':ab,ti	1817

#54	#51 OR #53	10573

**Intraoperative radiotherapy for pancreatic cancer	662**

#55	#26 AND #54	662

**Cochrane: 25**

**Keywords for pancreatic cancer	4557**

#1	MeSH descriptor: [Pancreatic Neoplasms] explode all trees	1434

#2	(Neoplasm*, Pancrea*):ti,ab,kw	0

#3	(Pancrea* Neoplasm*):ti,ab,kw	2258

#4	(Cancer*, Pancrea*):ti,ab,kw	0

#5	(Pancrea* Cancer*):ti,ab,kw	3586

#6	(Cancer of Pancreas):ti,ab,kw	1982

#7	(Cancer of the Pancreas):ti,ab,kw	1708

#8	#1 OR #2 OR #3 OR #4 OR #5 OR #6 OR #7	4246

#9	(Pancrea* carcinoma):ti,ab,kw	962

#10	(Pancrea* adenocarcinoma):ti,ab,kw	1159

#11	(Pancrea* malignant tumor):ti,ab,kw	366

#12	#8 OR #9 OR #10 OR #11	4557

**Keywords for radiotherapy	18555**

#13	MeSH descriptor: [Radiotherapy] explode all trees	5736

#14	(Radiation Therap*):ti,ab,kw	14036

#15	(Therap*, Radiation):ti,ab,kw	0

#16	(Radiation Treatment*):ti,ab,kw	12121

#17	(Treatment, Radiation):ti,ab,kw	12117

#18	(Radiotherap*, Targeted):ti,ab,kw	0

#19	(Targeted Radiotherap*):ti,ab,kw	0

#20	(Radiation Therap*, Targeted):ti,ab,kw	0

#21	(Targeted Radiation Therap*):ti,ab,kw	1154

#22	(Therap*, Targeted Radiation):ti,ab,kw	0

#23	#13 OR #14 OR #15 OR #16 OR #17 OR #18 OR #19 OR #20 OR #21 OR #22	18555

**Keywords for Intraoperative	19676**

#24	MeSH descriptor: [Intraoperative Period] explode all trees	2237

#25	(Intraoperative):ti,ab,kw	18634

#26	(intra-operative):ti,ab,kw	2077

#27	#24 OR #25 OR #26	19676

**intraoperative radiotherapy	369**

#28	#23 AND #27	333

#29	(IORT):ti,ab,kw	112

#30	#28 OR #29	369

**Intraoperative radiotherapy for pancreatic cancer	25**

#31	#30 AND #12	25
